# Assessing the cardiovascular effects of levothyroxine use in an ageing United Kingdom population (ACEL-UK) protocol: a cohort and target trial emulation study

**DOI:** 10.1186/s13044-023-00186-0

**Published:** 2023-11-13

**Authors:** Mia Holley, Salman Razvi, Rosie Dew, Ian Maxwell, Scott Wilkes

**Affiliations:** 1 School of Medicine, Faculty of Health Sciences and Wellbeing, University of Sunderland, Sunderland, UK; 2 Translational and Clinical Research Institute, Newcastle University, Newcastle-upon-Tyne, UK

**Keywords:** Aged, Cardiovascular, General practice, Hypothyroidism, Levothyroxine, Thyroid stimulating hormone, Thyroxine, Osteoporosis

## Abstract

**Background:**

Subclinical hypothyroidism is diagnosed when serum thyroid stimulating hormone levels are higher whilst free thyroxine levels remain within their respective reference ranges. These reference ranges are uniformly applied in all adults, despite serum thyroid stimulating hormone levels naturally increasing with age. Research has found that mildly elevated thyroid stimulating hormone levels may be associated with some benefits in ageing patients, including reduced mortality and better cardiorespiratory fitness. Levothyroxine is typically prescribed to patients with hypothyroidism, but no conclusive evidence exists on whether levothyroxine therapy is beneficial or detrimental in older subclinical hypothyroid patients. Despite this, prescriptions for levothyroxine are increasing year-on-year. This study aims to determine if receiving levothyroxine affects the cardiovascular and bone health outcomes of subclinical patients in primary care aged 50 years and over.

**Methods:**

This project includes a retrospective cohort analysis and a target trial emulation study using electronic patient records collected between 2006 and 2021 and recorded in The Health Improvement Network database. The primary outcome of this study is to compare the cardiovascular outcomes of subclinical hypothyroid patients aged over 50 years treated with levothyroxine compared to those untreated. Secondary outcomes are bone health and all-cause mortality outcomes. Descriptive and inferential statistics will both be employed to analyse the data. Secondary analysis will explore confounding factors, including age, sex, smoking status, body mass index, co-morbidities, and levothyroxine dosage.

**Discussion:**

There needs to be a greater understanding of the potential risks of the current treatment for older patients with subclinical hypothyroidism in a primary care setting. We will investigate the clinical importance of this issue and whether older subclinical hypothyroid patients have poorer outcomes when treated. Clarifying this concern may help address the healthcare resource implications of ageing patients being misclassified as having mild hypothyroidism, as these patients are more likely to repeat their blood tests. This could reduce prescription wastage and improve patient outcomes and quality of life in the ageing population.

**Trial registration:**

Not applicable.

## Background

Hypothyroidism is a common chronic disease caused by insufficient production of thyroid hormones [1]. Thyroid hormones produced by the thyroid gland regulate metabolism in all tissues, including the heart, liver, brain, muscles, and bones. Any interruption of the thyroid hormone balance can lead to metabolic dysfunction. The overall prevalence of hypothyroidism is approximately 5–10% of the general population in the United Kingdom (UK) [2] and is greater in females and people aged over 60 years [3–5]. Subclinical hypothyroidism (SCH) refers to serum thyroid stimulating hormone (TSH) levels higher than the reference range and levels of free thyroxine (fT4) remaining in the range [1, 6]. Overt hypothyroidism refers to TSH levels being elevated and fT4 levels below their respective reference ranges [7].

Cardiovascular disease remains the leading cause of death worldwide, with ischaemic heart disease and stroke accounting for 8.9 million and 6.1 million deaths, respectively, in 2019 [8]. Each year there are 160,000 deaths in the UK attributed to cardiovascular events [9], accounting for approximately 23.9% of deaths in the UK [10]. Therefore, unnecessary cardiovascular events must be reduced in the population. In addition, the UK population is increasing and ageing; the Office for National Statistics has predicted that by 2043, the number of people aged over 85 will have nearly doubled to 3 million [11]. Due to the ageing and growing population in the UK, a rise in pharmaceutical prescribing can be expected. In 2019, the National Health Service (NHS) distributed 32,956,754 prescriptions of LT4. This is a significant increase compared to only 20,426,378 LT4 prescriptions distributed in 2008 [12]. Moreover, the estimated number of individuals prescribed LT4 in the UK has increased from 2.8 to 4.1% over 11 years. Reducing the number of patients prescribed LT4 will not only potentially improve patient outcomes and quality of life in the ageing population but will reduce prescription wastage, hence cost to the NHS.

Mildly elevated TSH levels become more prevalent as people age [13]. The National Health and Nutrition Examination Survey, based in the United States (US), examined 16,533 people who were carefully selected to represent the general US population without thyroid disease and demonstrated that a significant proportion of older adults had high serum TSH (> 4.5 mIU/L) [4]. In addition, the Thyroid Epidemiology, Audit, and Research Study (TEARS) found that, based on a population of 153,127 people in Scotland, the 97.5th centile for TSH concentration steadily rose within age deciles, to an upper limit of 5.9 mIU/L in the over 90-year-olds [13]. This suggests that it is normal for the elderly population to have a TSH level within the range of 0.4–5.9 mIU/L rather than within the range of 0.4–4.0 mIU/L that is typically used. Furthermore, the longitudinal all-stars study, on 843 participants not taking thyroid medication, indicated that the 97.5th percentile TSH concentration could be as high as 8mIU/L in over 90-year-olds [14]. Longitudinal studies have also shown increased TSH levels in the same individuals over time, with no significant changes in fT4 concentrations [14–16]. Therefore, there is significant evidence to suggest that TSH concentration rises with age.

Slightly elevated TSH levels may be beneficial to the older person. Simonsick et al. [17] demonstrated better mobility and cardiorespiratory fitness functioning amongst adults over 70 years, with a mildly elevated TSH between 4.5 and 7.0 mIU/L compared to those with TSH levels of 0.4–4.5 mIU/L [17]. In the Leiden 85 + Study, 599 participants over 85 years old were followed up after four years. Those with a TSH level greater than 4.8 mIU/L had lower mortality than euthyroid individuals with a TSH level within 0.3–4.8 mIU/L; higher levels of fT4 were associated with increased all-cause mortality in the same cohort [18]. On the contrary, the Newcastle 85 + study found no association between higher TSH levels and all-cause mortality in a cohort of 643 patients aged 85 years followed up for 9 years [19]. In addition, a recent narrative review in 2021 found no unfavourable cardiovascular or musculoskeletal outcomes in patients with TSH levels between 4.5 and 7.0 mIU/L [6]. However, a case-control study found a significant mortality increase in patients prescribed LT4 aged over 65 with TSH below 10 mIU/L [20]. Much of the literature, including reviews that are powerful due to tertiary data, suggests that TSH levels between 4.5 and 10 mIU/L may benefit older adults.

However, TSH levels above 10 mlU/L have been found to be detrimental in all patients. A systematic review of 36 studies found an increased incidence of heart failure amongst patients aged over 65 years with TSH levels above 10 mlU/L [21]. Similarly, another systematic review of cohort studies found that in individuals with SCH and TSH levels above 10 mIU/L, there is an increased risk of coronary heart disease events, coronary heart disease mortality and heart failure events [22]. Current evidence indicates that TSH levels above 10 mlU/L can adversely affect older individuals. This is in line with the LT4 prescribing guidelines, stating that the majority of adults should only be prescribed LT4 with TSH levels above 10 mlU/L [23].

Previous studies that addressed the link between cardiovascular outcomes and LT4 treatment have failed to find a conclusive outcome. A cohort study on 1,642 patients aged over 70 years found no difference in cardiovascular events between treated and untreated groups [24]. The Thyroid Hormone Therapy for Older Adults with Subclinical Hypothyroidism (TRUST) study, a double-blind randomised placebo-controlled parallel-group trial of 737 adults aged 65 years and older with SCH, found no association between LT4 use and cardiovascular outcomes (hazard ratio 0.89; 95% confidence interval 0.47–1.69) [25]. The TRUST group concluded that LT4 should not be routinely prescribed to older people with SCH. Pooled results of the TRUST study and another randomised control trial found no significant difference in the cardiovascular risk of patients treated with LT4 versus those untreated [26]. However, both randomised controlled trials were underpowered due to small sample sizes. Most studies in older populations have not shown a clear association between SCH and cardiovascular events in patients aged over 65. The effect of mild thyroid failure on cardiovascular health becomes less evident with increasing age [27]. There is need for further large-scale studies investigating the impact of LT4 on cardiovascular health in patients over 50, particularly those aged between 50 and 65 years.

It is accepted that patients with overt hypothyroidism should be prescribed LT4 due to the association between the untreated condition and increased morbidity and mortality [28, 29]. However, the treatment of SCH is unclear due to a lack of reliable evidence in the past [30]. Over-treatment with thyroid hormones harms health, including poorer cardiovascular outcomes, atrial fibrillation, fractures, and osteoporosis [27, 31, 32]. However, over-treatment is common in older people and is associated with a longer time on replacement therapy and increasing age [33–35]. The European Thyroid Association guidelines recommend that SCH patients be prescribed LT4 when symptoms are shown and a TSH level is above 10 mIU/L [23]. It is also recommended that LT4 doses are personalised according to age, co-morbidities, and life expectancy [6]. However, research proves that these recommendations are not always followed [33].

The current literature suggests that older patients with mildly raised TSH concentrations may benefit from not being prescribed LT4 medication. The evidence is increasing and compelling to introduce age-specific TSH reference ranges for hypothyroidism regarding diagnostic thresholds and treatment targets to avoid patients being misclassified as having SCH and inappropriate treatment with thyroid hormones [13–15, 36, 37]. The distribution of TSH levels at the time of LT4 initiation has been described by Taylor et al., 2014 [33]. The median TSH when a patient is prescribed thyroid hormones has decreased from 8.7 mIU/L in 2001 to 7.9 mIU/L in 2009 in the UK, suggesting the likelihood a patient is prescribed LT4 treatment is increasing [33]. However, there has been a rising debate and emerging evidence suggesting that assessing borderline thyroid dysfunction may be more accurately characterised by fT4 levels rather than relying solely on TSH measurements [38, 39].

The literature is conflicting surrounding LT4 use and cardiovascular and bone health risk in the older population. A questionnaire answered by 408 individuals with SCH and taking LT4 found an increased risk of fractures in individuals over 50 years, particularly in the forearm [41]. It has also been found that the over-treatment of LT4 can increase the risk of cardiovascular morbidity, cardiovascular mortality and osteoporosis [42]. There is also conflicting evidence between the use of LT4 and mortality. The recent Baltimore Longitudinal Study of Ageing in 2021 found no association between all-cause mortality and LT4 use in over 65 year olds with normal TSH levels [43], while a much larger database study on 2,007,528 patients found that all-cause mortality was significantly higher in LT4-treated patients [44]. This suggests that LT4-based outcomes are dependent on both age and TSH levels.

The current research group has completed a National Institute for Health and Care Research (NIHR) Research for Patient Benefit study (Study of Optimal Replacement of Thyroxine in ElDerly (SORTED) – a feasibility study: Ref: PB-PG-0610-22139) and has demonstrated that older people can tolerate a lower dose of LT4 and hence higher TSH levels [45]. The work done in 2016 explored the relationship between the three variables: diagnosis of hypothyroidism, TSH measurement, and LT4 treatment. A feasibility analysis at one large rural general practice in Northumberland, England, with a practice list size of 11,752 found that 465 patients had been diagnosed with hypothyroidism. The prevalence of hypothyroidism at the practice was 4%, and the highest prevalence was in the 81–90 years age category, at 9.85%. The female-to-male ratio with hypothyroidism at the practice was 4.4:1.0, such that 379 females had been diagnosed. Despite 465 patients diagnosed with hypothyroidism (Read Coded), 622 patients received treatment with LT4. This is an excess of 157 patients receiving LT4 without a diagnosis of hypothyroidism. This may represent a proportion of SCH patients that are not Read Coded as hypothyroid but are receiving treatment. It is also possible that a small proportion of these patients are hyperthyroid patients receiving block and replace treatment or patients with pituitary disorders. Alternatively, some patients may report symptoms suggestive of hypothyroidism but still have normal serum thyroid function tests and receive LT4 therapy in contravention of accepted clinical practice and guidelines. For this reason, our search strategy centres upon the TSH values recorded in the database rather than a diagnosis. Between 2003 and 2015, 215 (1.9%) patients were recorded as having an age-specific index TSH as mildly elevated, in line with the TEARS study. Of those, 39% were untreated, and 61% were treated with varying degrees of treatment length. This feasibility work concludes that 53% of the treated patients may have been over-diagnosed due to their TSH level being within the range of 4.0 mIU/L and the upper TSH limit given by the TEARS study [13].

## Methods/Design

### Study aims

The study aims to evaluate whether LT4 benefits patients aged over 50 years with marginally raised TSH. The null hypothesis is that SCH patients over 50 years of age have the same bone health and cardiovascular outcomes when prescribed LT4 compared to those not prescribed LT4. The alternative hypothesis is that SCH patients aged over 50 years of age have worse bone health and cardiovascular outcomes when prescribed LT4 than those who are not prescribed LT4.

The objectives of the study are:

1) To use electronic healthcare records to undertake a cohort study comparing the cardiovascular outcomes (incident diagnosis of angina, myocardial infarction, stroke, peripheral vascular disease and stent/revascularisation procedure) between patients over 50 years of age with mildly elevated TSH levels prescribed LT4 versus those not prescribed LT4.

2) To use electronic healthcare records to undertake a cohort study comparing the bone health outcomes (incident diagnosis of osteoporosis, fragility fractures) between patients over 50 years of age with mildly elevated TSH levels prescribed LT4 versus those not prescribed LT4.

3) To use electronic healthcare records to undertake a cohort study comparing all-cause mortality outcomes between patients over 50 years of age with mildly elevated TSH levels prescribed LT4 versus those not prescribed LT4.

4) To use electronic healthcare records to undertake a target trial emulation study comparing the cardiovascular outcomes (incident diagnosis of angina, myocardial infarction, stroke, peripheral vascular disease and stent/revascularisation procedure) between patients over 50 years of age with mildly elevated TSH levels prescribed LT4 versus those not prescribed LT4 [46].

5) To use electronic healthcare records to undertake a target trial emulation study comparing the bone health outcomes (incident diagnosis of osteoporosis, fragility fractures) between patients over 50 years of age with mildly elevated TSH levels prescribed LT4 versus those not prescribed LT4.

6) To use electronic healthcare records to undertake a target trial emulation study comparing all-cause mortality outcomes between patients over 50 years of age with mildly elevated TSH levels prescribed LT4 versus those not prescribed LT4.

### Study design

A retrospective cohort study and a target trial emulation study will be conducted using electronic health records held in The Health Improvement Network (THIN) database.

### Study setting

THIN captures clinical data from general practices across the UK, holding anonymised records of approximately 6% of the UK population from 850 general practices. THIN has over 25 years of data recorded. To ensure a complete and reliable dataset, this project will capture data between January 1st, 2006, and December 31st, 2021. Preliminary counts from THIN show 530,792 patients with a TSH greater than 4 mIU/L. Preliminary counts also show 258,868 patients diagnosed with hypothyroidism within the same period. The variables that will be captured from the THIN database are as follows:

- sex,
- diagnosed with hypothyroidism,
- date of diagnosis of hypothyroidism,
- date LT4 treatment commenced,
- first and last recorded LT4 dose,
- co-morbidities (heart disease, asthma, chronic obstructive pulmonary disease, diabetes, chronic kidney disease, rheumatoid arthritis, depression, dementia, dyslipidaemia, osteoporosis, fragility fractures).
- date of diagnosis of co-morbidities,
- current and past medications,
- date of death (if applicable),
- TSH, fT4, thyroxine (T4), triiodothyronine (T3), and free triiodothyronine (fT3) levels,
- date of recorded TSH, fT4, T4, T3, and fT3 levels,
- thyroid peroxidase antibody status,
- body mass index (BMI) at entry and end of the study period,
- blood pressure,
- smoking status.

#### Inclusion criteria

1. Patients registered on the THIN database and aged over 50 on January 1st, 2006.
2. Patients with a recording of TSH above 4.0 mIU/L between January 1st, 2006, and December 31st, 2021.
3. Patients who are prescribed LT4 or receive no hormone replacement therapy. In the cohort study, patients in the treatment group will be prescribed LT4 before the end of the study period, December 31st, 2021. In the emulated target trial study, patients will initiate LT4 treatment and remain on it during a 5-year follow-up between January 1st, 2006, and December 31st, 2021. Patients in the emulated target trial study will be randomly assigned a strategy at baseline and be aware of their assignment.
4. In the cohort study, patient follow-up ends at death, withdrawal from the database or December 31st, 2021, whichever occurs first.

#### Exclusion criteria

1. Patients aged 50 years or under on January 1st, 2006.
2. Patients without a recording of TSH above 4.0 mIU/L between January 1st, 2006, and December 31st, 2021.
3. Patients diagnosed with thyroid cancer, pituitary disease or hyperthyroidism.
4. Patients prescribed medications such as amiodarone or lithium that can affect thyroid function.
5. Patients who have undergone thyroid surgery or radioiodine treatment, suggesting previous thyroid dysfunction.
6. Patients receiving a different form of thyroid replacement therapy to LT4, e.g. liothyronine or combination therapy with liothyronine and LT4.
7. Patients with a baseline diagnosis of angina, myocardial infarction, peripheral vascular disease or stroke, where cardiovascular is the outcome variable.
8. Patients with a baseline diagnosis of osteoporosis or fracture, where bone health is the outcome variable.

### Statistical analysis

The sample size calculation is based on the primary outcome of determining a difference in cardiovascular outcomes between patients receiving LT4 or not. The TRUST study of 737 patients aged 65 years and older found a cardiovascular outcome rate of 4.9% in the treatment group and 5.4% in the placebo group. Therefore, based on these outcome rates, for the cohort study, the minimum sample size required is 82,118 patients. Of this sample, 41,059 patients will be prescribed LT4 and 41,059 patients will not be prescribed LT4. A matching ratio of 1:4 (treatment: no treatment) will be used for the target trial emulation study. Therefore, the sample size required is 129,020 patients, with 25,804 patients prescribed LT4 and 103,216 patients not prescribed LT4. This sample size provides a power of 0.9 and alpha of 5%. In both studies, if missing data occurs, multiple imputation methods will be used if up to 40% of the data is missing [47]. Otherwise, pairwise deletion will be used.

In the target trial emulation study, the causal contrasts of interest will be intention-to-treat effect. Chi-square analysis will be used to compare the categorical characteristics of the participants in both studies, such that a p-value of less than 0.05 will be considered significant. Descriptive statistics will be presented. In both studies, the frequency of hypothyroidism diagnosis, smoking status, sex, ethnicity, and location will be given for each group. Survival analysis methods, including Cox proportional hazard models and Kaplan-Meier curves, will be fitted to compare the time to outcome between the groups. The hazard ratios with their 95% confidence intervals will be given.

Confounding bias, immortal time bias, and information bias will be adjusted for both the cohort and target trial emulation studies [48]. In addition, selection bias and attrition bias will be adjusted for in the target trial emulation study. Confounding factors include sex, BMI, co-morbidities, and smoking status. The adjustment for confounding factors will be done using inverse probability weighting in the target trial emulation study [49]. In both studies, subgroup analysis will be done for each cardiovascular and bone health outcome and each age group (51–60, 61–70, 71–80, 81–90, 91 + years). A subgroup analysis including patients with a TSH level between 4 0.0 mIU/L and the upper limit of their age range in the TEARS study will be carried out. Another subgroup analysis of patients who have had a TSH level of 10 mIU/L or above or not will be conducted. Also, sensitivity analysis will be undertaken in both studies for different TSH thresholds, as the standard threshold can differ between laboratories. This study will perform a secondary analysis based on baseline fT4 levels, comparing patients with a low normal versus high normal levels (below and above median levels at baseline, respectively).

## Discussion

The ACEL-UK study will add weight to the body of knowledge on this critical issue for ageing patients who are potentially experiencing significant harm from the possible over-prescription of thyroid hormones and will provide data to support or refute the concept of increased risk of cardiovascular and bone health risk in ageing patients. This study will look at LT4 treatment in ageing patients with mildly elevated TSH levels. The outcomes of this study may have implications for clinical practice and prescribing LT4 in the UK. Suggestions for future research after this study will include a randomised control trial with a large sample size to confirm the results of this study.

The main outputs will be reports (funder, local and national policymakers), academic publications, and national and international conference presentations. We will compile a summary report for publication in the Journal of Health Services Research to reach the NHS Commissioning Readership. We will disseminate our findings in popular GP press and peer-reviewed primary care and endocrine journals.

## Data Availability

The data that supports the finding of this study are available from THIN a wholly owned subsidiary of Cegedim SA who own the proprietary rights in THIN data. Restrictions apply to the availability of these data, which were used under license for the current study, and so are not publicly available. Data are however available from the authors upon reasonable request and with the permission of THIN. The datasets used during the feasibility study are available from the corresponding author on reasonable request.

## List of abbreviations

ACEL: UK Assessing the Cardiovascular Effects of Levothyroxine Use in an Ageing United Kingdom population
BMI: Body Mass Index
fT3: Free Triiodothyronine
fT4: Free Thyroxine
LT4: Levothyroxine
NHS: National Health Service
NIHR: National Institute for Health and Care Research
SCH: Subclinical Hypothyroidism
T3: Triiodothyronine
T4: Thyroxine
TEARS: Thyroid Epidemiology, Audit, and Research Study
THIN: The Health Improvement Network
TRUST: Thyroid Hormone Therapy for Older Adults with Subclinical Hypothyroidism
TSH: Thyroid Stimulating Hormone
UK: United Kingdom
US: United States

## References

[1] Cooper DS, Biondi B (2012). Subclinical thyroid Disease. The Lancet.

[2] Okosieme O, Gilbert J, Abraham P, Boelaert K, Dayan C, Gurnell M (2016). Management of primary hypothyroidism: statement by the British Thyroid Association Executive Committee. Clin Endocrinol (Oxf).

[3] Leese GP, Flynn RV, Jung RT, Macdonald TM, Murphy MJ, Morris AD (2008). Increasing prevalence and incidence of thyroid Disease in Tayside, Scotland: the thyroid epidemiology audit and Research Study (TEARS). Clin Endocrinol (Oxf).

[4] Hollowell JG, Staehling NW, Flanders WD, Hannon WH, Gunter EW, Spencer CA (2002). Serum TSH, T(4), and thyroid antibodies in the United States population (1988 to 1994): National Health and Nutrition Examination Survey (NHANES III). J Clin Endocrinol Metab.

[5] Parle JV, Franklyn JA, Cross KW, Jones SC, Sheppard MC (1991). Prevalence and follow-up of abnormal thyrotrophin (TSH) concentrations in the elderly in the United Kingdom. Clin Endocrinol (Oxf).

[6] Biondi B, Cappola AR (2022). Subclinical hypothyroidism in older individuals. LANCET DIABETES Endocrinol.

[7] Biondi B, Cappola AR, Cooper DS (2019). Subclinical hypothyroidism: a review. JAMA.

[8] The top 10. causes of death [Internet]. [cited 2021 Nov 26]. Available from: https://www.who.int/news-room/fact-sheets/detail/the-top-10-causes-of-death.

[9] British Heart Foundation [Internet]. [cited 2021 Nov 29]. Heart Statistics. Available from: https://www.bhf.org.uk/what-we-do/our-research/heart-statistics.

[10] Population estimates for the UK., England and Wales, Scotland and Northern Ireland - Office for National Statistics [Internet]. [cited 2021 Nov 29]. Available from: https://www.ons.gov.uk/peoplepopulationandcommunity/populationandmigration/populationestimates/bulletins/annualmidyearpopulationestimates/mid2020.

[11] National population projections - Office for National Statistics [Internet]. [cited 2021 Nov 26]. Available from: https://www.ons.gov.uk/peoplepopulationandcommunity/populationandmigration/populationprojections/bulletins/nationalpopulationprojections/2018based#changes-since-the-2016-based-projections.

[12] Robinson J. The Pharmaceutical Journal. [cited 2022 Nov 1]. Prescribing of thyroid hormone increases by more than 60% in a decade. Available from: https://pharmaceutical-journal.com/article/news/prescribing-of-thyroid-hormone-increases-by-over-60-in-a-decade.

[13] Vadiveloo T, Donnan PT, Murphy MJ, Leese GP (2013). Age- and gender-specific TSH reference intervals in people with no obvious thyroid Disease in Tayside, Scotland: the thyroid epidemiology, audit, and Research Study (TEARS). J Clin Endocrinol Metab.

[14] Waring AC, Arnold AM, Newman AB, Bùzková P, Hirsch C, Cappola AR (2012). Longitudinal changes in thyroid function in the oldest old and survival: the cardiovascular health study all-stars study. J Clin Endocrinol Metab.

[15] Bremner AP, Feddema P, Leedman PJ, Brown SJ, Beilby JP, Lim EM (2012). Age-related changes in thyroid function: a longitudinal study of a community-based cohort. J Clin Endocrinol Metab.

[16] Chaker L, Korevaar TIM, Medici M, Uitterlinden AG, Hofman A, Dehghan A (2016). Thyroid function characteristics and determinants: the Rotterdam Study. Thyroid®.

[17] Simonsick EM, Newman AB, Ferrucci L, Satterfield S, Harris TB, Rodondi N (2009). Subclinical hypothyroidism and functional mobility in older adults. Arch Intern Med.

[18] Gussekloo J, van Exel E, de Craen AJM, Meinders AE, Frölich M, Westendorp RGJ (2004). Thyroid status, disability and cognitive function, and survival in old age. JAMA.

[19] Pearce SHS, Razvi S, Yadegarfar ME, Martin-Ruiz C, Kingston A, Collerton J (2016). Serum thyroid function, mortality and disability in Advanced Old Age: the Newcastle 85 + study. J Clin Endocrinol Metab.

[20] Grossman A, Feldhamer I, Meyerovitch J (2018). Treatment with levothyroxin in subclinical hypothyroidism is associated with increased mortality in the elderly. Eur J Intern Med.

[21] Panday P, Arcia Franchini AP, Iskander B, Anwer F, Oliveri F, Kakargias F (2021). Subclinical hypothyroidism in Geriatric Population and its Association with Heart Failure. Cureus.

[22] Gencer B, Collet TH, Virgini V, Auer R, Rodondi N (2013). Subclinical thyroid dysfunction and cardiovascular outcomes among prospective cohort studies. Endocr Metab Immune Disord Drug Targets.

[23] Pearce SHS, Brabant G, Duntas LH, Monzani F, Peeters RP, Razvi S (2013). 2013 ETA Guideline: management of subclinical hypothyroidism. Eur Thyroid J.

[24] Razvi S, Weaver JU, Butler TJ, Pearce SHS (2012). Levothyroxine treatment of subclinical hypothyroidism, fatal and nonfatal cardiovascular events, and mortality. Arch Intern Med.

[25] Ogunsakin A, Solomon SS, Dagogo-Jack S (2017). Thyroid hormone therapy for older adults with subclinical hypothyroidism. N Engl J Med.

[26] Zijlstra L, Jukema J, Westendorp R, Du Puy R, Poortvliet R, Kearney P et al. Levothyroxine Treatment and Cardiovascular Outcomes in Older People With Subclinical Hypothyroidism: Pooled Individual Results of Two Randomised Controlled Trials. Front Endocrinol [Internet]. 2021;12. Available from: https://www.cochranelibrary.com/central/doi/10.1002/central/CN-02285545/full. 10.3389/fendo.2021.674841 PMC8173189 34093444

[27] Biondi B, Cooper DS (2008). The clinical significance of subclinical thyroid dysfunction. Endocr Rev.

[28] Bensenor IM, Olmos RD, Lotufo PA (2012). Hypothyroidism in the elderly: diagnosis and management. Clin Interv Aging.

[29] Boelaert K, Franklyn JA (2005). Thyroid hormone in health and Disease. J Endocrinol.

[30] Biondi B, Wartofsky L (2014). Treatment with thyroid hormone. Endocr Rev.

[31] Flynn RW, Bonellie SR, Jung RT, MacDonald TM, Morris AD, Leese GP (2010). Serum thyroid-stimulating hormone concentration and morbidity from Cardiovascular Disease and fractures in patients on long-term thyroxine therapy. J Clin Endocrinol Metab.

[32] Ko YJ, Kim JY, Lee J, Song HJ, Kim JY, Choi NK (2014). Levothyroxine dose and fracture risk according to the osteoporosis status in elderly women. J Prev Med Public Health Yebang Uihakhoe Chi.

[33] Taylor PN, Iqbal A, Minassian C, Sayers A, Draman MS, Greenwood R (2014). Falling threshold for treatment of borderline elevated thyrotropin levels-balancing benefits and risks: evidence from a large community-based study. JAMA Intern Med.

[34] De Whalley P (1995). Do abnormal thyroid stimulating hormone level values result in treatment changes? A study of patients on thyroxine in one general practice. Br J Gen Pract J R Coll Gen Pract.

[35] Somwaru LL, Arnold AM, Joshi N, Fried LP, Cappola AR (2009). High frequency of and factors associated with thyroid hormone over-replacement and under-replacement in men and women aged 65 and over. J Clin Endocrinol Metab.

[36] Surks MI, Hollowell JG (2007). Age-specific distribution of serum thyrotropin and antithyroid antibodies in the US population: implications for the prevalence of subclinical hypothyroidism. J Clin Endocrinol Metab.

[37] Boucai L, Surks MI (2009). Reference limits of serum TSH and free T4 are significantly influenced by race and age in an urban outpatient medical practice. Clin Endocrinol (Oxf).

[38] Fitzgerald SP, Bean NG, Falhammar H, Tuke J (2020). Clinical parameters are more likely to be Associated with thyroid hormone levels than with thyrotropin levels: a systematic review and Meta-analysis. Thyroid off J Am Thyroid Assoc.

[39] Fitzgerald SP, Bean NG, Hennessey JV, Falhammar H (2021). Thyroid testing paradigm switch from thyrotropin to thyroid hormones-future directions and opportunities in clinical medicine and research. Endocrine.

[40] Bornschein A, Paz-Filho G, Graf H, Carvalho GAD (2012). Treating primary hypothyroidism with weekly doses of levothyroxine: a randomized, single-blind, crossover study. Arq Bras Endocrinol Metabol.

[41] Vestergaard P, Weeke J, Hoeck HC, Nielsen HK, Rungby J, Rejnmark L (2000). Fractures in patients with primary idiopathic hypothyroidism. Thyroid off J Am Thyroid Assoc.

[42] Effraimidis G, Watt T, Feldt-Rasmussen U (2021). Levothyroxine Therapy in Elderly patients with hypothyroidism. Front Endocrinol.

[43] Abbey EJ, McGready J, Ferrucci L, Simonsick EM, Mammen JSR (2021). Thyroid hormone supplementation and all-cause Mortality in Community-Dwelling older adults: results from the Baltimore Longitudinal Study of Aging. J Am Geriatr Soc.

[44] Sohn SY, Seo GH, Chung JH (2021). Risk of all-cause mortality in levothyroxine-treated hypothyroid patients: a nationwide Korean cohort study. Front Endocrinol.

[45] Razvi S, Ingoe L, Ryan V, Pearce SHS, Wilkes S (2016). Study of optimal replacement of Thyroxine in the Elderly (SORTED) - results from the feasibility randomised controlled trial. Thyroid Res.

[46] Hernán MA, Robins JM (2016). Using Big Data to emulate a target Trial when a Randomized Trial is not available. Am J Epidemiol.

[47] Jakobsen JC, Gluud C, Wetterslev J, Winkel P (2017). When and how should multiple imputation be used for handling missing data in randomised clinical trials – a practical guide with flowcharts. BMC Med Res Methodol.

[48] Lévesque LE, Hanley JA, Kezouh A, Suissa S (2010). Problem of immortal time bias in cohort studies: example using statins for preventing progression of Diabetes. BMJ.

[49] Hernan MA, Robins JM. Causal Inference: What If.

